# LE-MDCAP: A Computational Model to Prioritize Causal miRNA-Disease Associations

**DOI:** 10.3390/ijms222413607

**Published:** 2021-12-19

**Authors:** Zhou Huang, Yu Han, Leibo Liu, Qinghua Cui, Yuan Zhou

**Affiliations:** Department of Biomedical Informatics, Ministry of Education Key Lab of Cardiovascular Sciences, School of Basic Medical Sciences, Peking University, Beijing 100191, China; ahuang_azhou@pku.edu.cn (Z.H.); sx_hanyu@bjmu.edu.cn (Y.H.); liuleibo_stu@163.com (L.L.); cuiqinghua@hsc.pku.edu.cn (Q.C.)

**Keywords:** miRNAs, diseases, miRNA-disease associations, causal miRNA-disease association prediction, Levenshtein distance

## Abstract

MicroRNAs (miRNAs) are associated with various complex human diseases and some miRNAs can be directly involved in the mechanisms of disease. Identifying disease-causative miRNAs can provide novel insight in disease pathogenesis from a miRNA perspective and facilitate disease treatment. To date, various computational models have been developed to predict general miRNA-disease associations, but few models are available to further prioritize causal miRNA-disease associations from non-causal associations. Therefore, in this study, we constructed a Levenshtein-Distance-Enhanced miRNA-disease Causal Association Predictor (LE-MDCAP), to predict potential causal miRNA-disease associations. Specifically, Levenshtein distance matrixes covering the sequence, expression and functional miRNA similarities were introduced to enhance the previous Gaussian interaction profile kernel-based similarity matrix. LE-MDCAP integrated miRNA similarity matrices, disease semantic similarity matrix and known causal miRNA-disease associations to make predictions. For regular causal vs. non-disease association discrimination task, LF-MDCAP achieved area under the receiver operating characteristic curve (AUROC) of 0.911 and 0.906 in 10-fold cross-validation and independent test, respectively. More importantly, LE-MDCAP prominently outperformed the previous MDCAP model in distinguishing causal versus non-causal miRNA-disease associations (AUROC 0.820 vs. 0.695). Case studies performed on diabetic retinopathy and hsa-mir-361 also validated the accuracy of our model. In summary, LE-MDCAP could be useful for screening causal miRNA-disease associations from general miRNA-disease associations.

## 1. Introduction

MicroRNAs (miRNAs) are a class of endogenous small RNAs of ~20 nucleotides in length that have various regulatory roles within cells. MiRNAs suppress target mRNA expression at the post-transcriptional level by binding to the 3′ untranslated regions (3′-UTRs) [1,2]. Accumulating evidence has demonstrated that miRNAs are involved in diverse biological processes, such as cell proliferation, differentiation, death and signal transduction [2,3,4]. Accordingly, more and more miRNAs have been confirmed to be associated with the onset and development of complex diseases [5]. For instance, miR-1 is dysregulated in multiple common heart disease [6,7], miR-355 and miR-31 are connected with the inhibition of breast cancer [8,9] and the loss of miR-206 accelerates amyotrophic lateral sclerosis (ALS) progression [10]. Therefore, the effective identification of miRNA-disease associations, especially miRNAs directly involved in disease mechanisms, is critical for promoting the treatment of complex human diseases.

With the growing body of research on the associations between miRNAs and diseases, 35,547 miRNA-disease association entries from a wide range of experimental evidence were gathered in the latest version of HMDD (v3.2, released in March 2019) [11]. Based on the type of experimental evidence, miRNA-disease associations can be sorted into causal associations (i.e., miRNAs that can be directly involved in disease mechanisms) and non-causal associations (i.e., miRNAs that exhibit differential expression but no known evidence of direct involvement in disease mechanisms) [12,13]. The causal miRNA-disease associations play a pivotal role in gaining insight into the molecular and cellular mechanisms of a disease and in identifying target miRNAs for further intervention. In the latest HMDD v3.2 database, Gao et al. [12] annotated causal associations by conducting a manual review of the literature. Specifically, in the “target” category of miRNA-disease associations, we selected the associations in which miRNAs target disease causal genes; meanwhile, in the “genetics” category of miRNA-disease associations, we selected the associations in which the genetic perturbation (knockdown/overexpression) of miRNAs could lead to altered disease phenotypes. Moreover, the associations in which miRNAs could enhance drug effects but have no contributions to diseases were excluded. Further manual confirmation by at least two investigators was performed, and, finally, 4294 miRNA-disease associations were labeled as causal associations. This sizable and biologically validated dataset enabled better investigations of general or even causal miRNA-disease associations by computational methods. Given the costly and time-consuming nature of traditional experimental methods, more and more researchers are using computational prediction models to effectively explore the relationship between miRNA and disease [14,15], such as label propagation algorithms used by MCLPMDA [16], LPNLS [17] and SNMDA [18]; machine-learning classification algorithms adopted by EGBMMDA [19]; and latent feature extraction with positive samples taken by LFEMDA [20], among others.

Although vast models have been designed to predict general disease-related miRNAs, our previous benchmark study has shown that most of these models could not distinguish causal miRNA-disease associations from non-causal associations (with AUROC < 0.55 in the task for causal/non-causal association discrimination) [12]. In other words, there is still an urgent demand for a new model specifically designed for prioritizing causal miRNA-disease association. To this end, Gao et al. [13] first proposed the MiRNA-disease Causal Association Predictor (MDCAP) based on the label propagation algorithm for predicting potential causal miRNA-disease associations. MDCAP showed reliable performance (AUCOR > 0.9) in distinguishing between causal miRNA-disease associations and unrelated miRNA-disease pairs. However, as for the discrimination between causal and non-causal miRNA-disease associations, the performance of MDCAP is greatly reduced.

In this study, we developed a novel prediction model named LE-MDCAP (Levenshtein-Distance-Enhanced MiRNA-disease Causal Association Predictor), based on Levenshtein distance and matrix decomposition algorithm, to prioritize potential causal miRNA-disease associations. The key improvement of LE-MDCAP is that it could specifically discriminating between causal and non-causal miRNA-disease associations, facilitating more precise identification of potential disease-causative miRNAs. To demonstrate the effectiveness of our proposed approach, we performed 10-fold cross-validation and independent tests to comprehensively measure our model performance. Moreover, the case study further validated the model reliability by comparing the prediction results with the latest experimental evidence that has not been considered in the HMDD v3.2 datasets.

## 2. Results

### 2.1. Overview of LE-MDCAP and Overall Performance Evaluation

**Figure 1:**
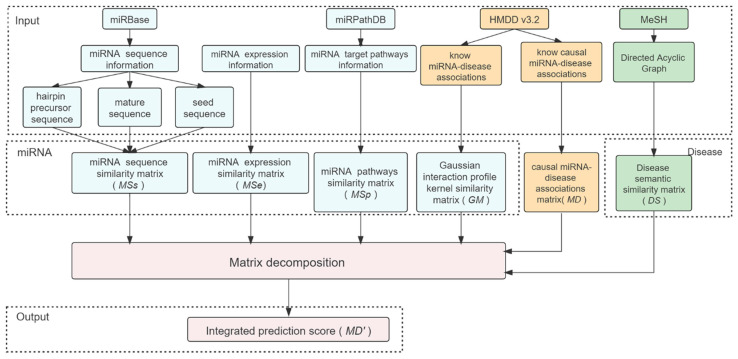
Flowchart of the LE-MDCAP prediction model.

In this work, we developed LE-MDCAP to predict potential causal miRNA-disease associations. Figure 1 shows the workflow of LE-MDCAP. First of all, we obtained a causal miRNA-disease association matrix with causal association data from HMDD v3.2. Second, we integrated multiple sources of information to represent miRNA similarity, including sequence similarity, expression level similarity and target pathway similarity, all calculated in the form of Levenshtein distances, in addition to Gaussian similarity. For diseases, the semantic similarity matrix was constructed based on the known disease relationships in MeSH. Finally, the matrix decomposition algorithm was introduced to establish the model based on each source of the miRNA similarity matrix, and the prediction scores from each model were integrated by using the weighted summing approach as the final prediction results of LE-MDCAP.

**Figure 2:**
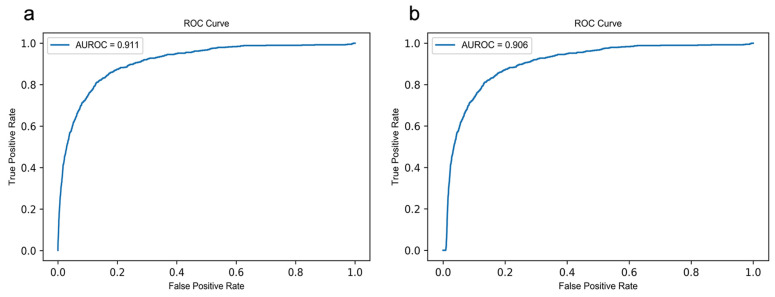
ROC curves performed by LE-MDCAP. (**a**) ROC curve of independent testing set. (**b**) ROC curve of 10-fold cross-validation.

To evaluate the overall performance of model LE-MDCAP, we performed 10-fold cross-validation and independent testing based on known causal miRNA-disease associations in HMDD v3.2. One-fifth of the known causal associations were randomly selected as the independent testing set, and the remaining four-fifths were used as the training set. Similarly, in each round of 10-fold cross-validation, the causal associations were divided into a training set and a test set in proportion. To avoid leakage of test data, the prediction results of the model were solely calculated based on the training set. Then, we plotted receiver operating characteristic (ROC) curves by calculating the true positive rate (TPR) and false positive rate (FPR) at different thresholds and calculated the area under the ROC curve (AUROC). The closer the AUROC value is to 1, the better the predictive effect of the prediction model. As shown in Figure 2, LE-MDCAP obtained AUROC values of 0.911 in the 10-fold cross-validation on the training dataset, and a comparable performance of AUROC = 0.906 was achieved in the independent testing. These results demonstrated the better performance of our method in predicting potentially causal miRNA-disease associations.

**Figure 3:**
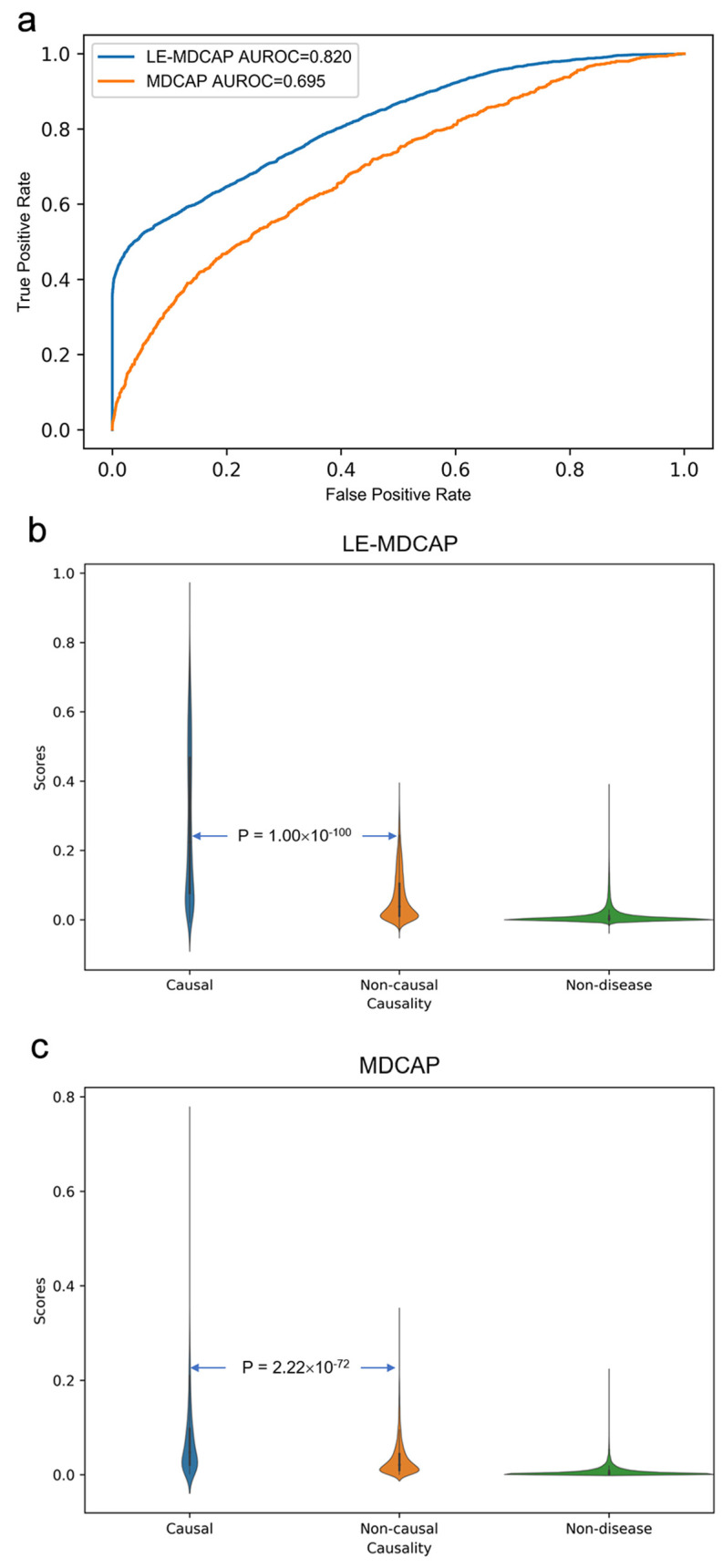
Performance comparisons between LE-MDCAP and MDCAP for prioritizing disease causal miRNAs. (**a**) ROC curves performed by LE-MDCAP and MDCAP in distinguishing causal miRNA-disease associations from the non-causal miRNA-disease associations. (**b**,**c**) Violin plots of LE-MDCAP and MDCAP showing the distribution of model prediction scores in the three groups.

Because such causal associations versus non-associations can also be well distinguished by the previous MDCAP model, we here focused on the reliability of the model in distinguishing causal and non-causal miRNA-disease associations, where performance of the previous MDCAP model was largely compromised. To this end, we divided all miRNA-disease pairs in the dataset into three groups as causal miRNA-disease associations (causal), non-causal miRNA-disease associations (non-causal) and unassociated miRNA-disease pairs (non-disease). We chose for these causal miRNA-disease associations to be the positive samples and non-causal miRNA-disease associations to be the negative samples for method evaluation. It is noteworthy that, to justify the method comparison, the prediction results from the above-described model were directly used on this dataset, and no model retraining was conducted during this evaluation. Indeed, the previous MDCAP model did not distinguish the causal versus non-causal group with a satisfactory accuracy (AUROC = 0.695). By contrast, as shown in Figure 3a, LE-MDCAP shows a unique advantage in discriminating causal and non-causal miRNA-disease associations (AUROC = 0.820). We also assessed the statistical significance of the difference in the prediction scores between three miRNA-disease groups (i.e., causal, non-causal and non-disease) by the Wilcoxon rank sum test. Figure 3b,c shows an increasing tendency from the prediction scores in the non-disease group to those in the causal group. Moreover, there is a significant difference between the causal and non-causal prediction scores in the LE-MDCAP prediction scores (*p* = 1.00 × 10^−100^), and this difference is more pronounced than in the MDCAP (*p* = 2.22 × 10^−72^). Together, the stepped distribution of prediction scores between the three groups suggests that LE-MDCAP can identify causal associations not only from a large number of unassociated miRNA-disease pairs, but also further efficiently from non-causal associations.

We have also tried to include a new feature in the model based on the target gene relationship of miRNAs with disease genes in order to further improve the predictive performance of LE-MDCAP. While its performance in identifying causal and non-causal miRNA disease associations did not improve, the addition of the new target gene features improved the model’s AUROC from 0.901 to 0.907 in distinguishing causal miRNA disease associations from unrelated miRNA disease pairs (Supplementary Materials Figure S1). Since the improvement is still marginal, we did not include target gene features in our final model. However, this result suggests that miRNA-gene-targeting features would be a possible direction for the future improvements of disease-causative miRNA predictions.

### 2.2. Case Study

To further verify the effectiveness of LE-MDCAP, we implemented case studies on causal miRNA-disease associations with high prediction scores by querying the latest literature records that have not been included in the HMDD v3.2 dataset. Because these articles were not included in either the training or the testing dataset, they could serve as a supplementary evaluation of LE-MDCAP’s performance in addition to the regular ROC assessments. We first checked if the LE-MDCAP prediction would facilitate finding the potential causal miRNAs of the investigated diseases. Diabetic retinopathy is a common and specific microvascular complication of diabetes that can lead to blindness in severe cases [21]. Understanding the molecular mechanisms of diabetic retinopathy can help develop therapeutic agents to alleviate the symptoms. Here, we looked for miRNAs with causal potential for diabetic retinopathy based on the predictive score of LE-MDCAP. As shown in Table 1, four of the top five and eight of the top 15 causal miRNA-disease associations in prediction scores have been validated by the literature in the last two years. We found that hsa-mir-21 had a score of 0.104, ranking the best among all potential miRNAs. Moreover, the upregulation of hsa-miR-21-5p has been reported to damage human retinal pigment epithelial cells, thereby inducing the development of diabetic retinopathy [22]. Another research found that hsa-miR-34a promotes vascular endothelial cell apoptosis in diabetic retinopathy by targeting SIRT1 [23]. Similarly, a study showed that hsa-miR-221-3p regulates microvascular dysfunction in diabetic retinopathy by targeting TIMP3 [24]. Moreover, a study published in the last year reported that has-miR-126 enhances proliferation and inhibits apoptosis in high-glucose-induced human retinal endothelial cells by targeting IL-17A, which in turn accelerates the disease process [25]. These articles were all published recently and have not yet been included in the HMDD v3.2 database, so they will not affect the prediction results of the LE-MDCAP algorithm. In general, it can be confirmed that the associations between potentially causal miRNAs predicted by LE-MDCAP and diabetic retinopathy are indeed causal.

In addition, we also checked if LE-MDCAP can facilitate identifying potential causal-associated diseases of a given miRNA. Here we selected has-mir-361 as a case study to verify the performance of our model. Previous studies have shown that hsa-mir-361 plays a crucial role in the development of several cancer types and cardiovascular diseases [26,27]. Excluding diseases in the training set for which causality have been identified, we used LE-MDCAP to predict other causal disease associations for hsa-mir-361. The prediction results are shown in Table 2, five out of the top five and ten out of the top 15 on the list were verified based on recent experimental reports. We also found a score of 0.157 for breast neoplasms, which ranked the best among all potential diseases. A study on breast neoplasms and hsa-mir-361 indicated that has-miR-361-3p promotes human breast cancer cell viability by inhibiting the E2F1/P73 signaling pathway [28]. Hsa-miR-361-5p was reported to exert tumor-suppressing functions in gastric carcinoma by targeting syndecan-binding protein [29]. Furthermore, long non-coding RNA BLACAT1 inhibits cell proliferation in prostate cancer by acting on hsa-miR-361 [30]; long non-coding RNA PVT1 contributes to cell growth and metastasis in non-small-cell lung cancer by regulating miR-361-3p [31]. Glioblastoma-related studies confirm that COX10-AS1 competitively binds hsa-mir-361-5p to promotes glioma development [32]. All of the above experimental investigations have suggested that hsa-mir-361 is involved in the progression of the disease predicted by LE-MDCAP. Taken together, the results of the analysis further confirmed the capability of LE-MDCAP to predict causal miRNA-disease associations.

### 2.3. LE-MDCAP Server

To facilitate the community, we established an easy-to-query webserver interface for LE-MDCAP (http://www.rnanut.net/LEMDCAP/). The query interface of the LE-MDCAP server is shown in Supplementary Materials Figure S2. The users can retrieve prediction results based either on a miRNA name keyword or a disease-term keyword. Bothe exact and fuzzy searching mode were supported. The users can also customize the method to sort the prediction scores, according to the per miRNA ranking (miRNA), the per disease ranking (disease, default) or the overall ranking (any), so that the most likely causative miRNA in the specific diseases can be easily prioritized. We also provided the dataset and all prediction results at the more stable GitHub Repository (https://github.com/bioinfohy/LE-MDCAP/), as an alternative data-access approach.

## 3. Discussion

As miRNA research has expanded into a large number of disease areas, it has become clear that the expression levels of certain miRNAs are altered in many diseases. Most of these miRNAs are only passively altered during disease progression, and we refer to these miRNA-disease associations as non-causal associations. Although disease non-causal miRNAs are not directly involved in disease mechanisms, they are widely employed in clinical diagnosis, treatment response and prognosis, due to their sensitivity. Evidence suggests that they can play an important role as biomarkers in cancer through exosome-mediated intercellular communication [33,34] and in neurology for the diagnosis and prognosis of Alzheimer’s disease [35], among others. However, for the purpose of accurate dissection of disease mechanisms or effective identification of therapeutic targets of miRNA interventions, causal miRNA-disease associations are more important.

Many algorithms have been proposed to screen miRNA-disease associations, but few of them have considered the more critical causal information during disease progression. Gao et al. proposed a model MDCAP for predicting causal miRNA-disease associations based on the latest disease causality annotation from HMDD v3.2 [13]. Nevertheless, as shown above, MDCAP cannot effectively discern between causal and non-causal miRNA-disease associations. Therefore, we constructed LE-MDCAP, a model for predicting causal miRNA-disease associations by using Levenshtein distance and matrix decomposition algorithms as a framework. LE-MDCAP exhibits competitive performance in both a 10-fold cross-validation and independent test. Notably, LE-MDCAP showed considerable advantages over MDCAP in prioritizing disease causal miRNAs from non-causal ones, highlighting the unique advantages for distinguishing between causal and non-causal miRNA-disease associations. The contribution of Levenshtein-distance-based similarity is intuitively expressed by the weights from the optimized prediction score integration formula as follows: *MD’ =* 0.35*MD’_S_ +* 0.4 *MD’_E_ +* 0.15*MD’_P_ +* 0.1*MD’_G_*. The Gaussian interaction profile kernel similarity matrix, which is the core similarity matrix of the previous MDCAP model, only contributes a minor fraction to the final prediction result (the weight of *MD’_G_* is only 0.1). By integrating similarity in the miRNA seed sequences, mature miRNA sequences and pre-miRNA sequences, the sequence-based Levenshtein-distance similarity matrix becomes a core component of the model (with the weight of *MD*’*_S_* being 0.35). Moreover, further integration of expression- and pathway-based Levenshtein-distance similarities also significantly contribute to the final model (the weights are 0.4 and 0.15, respectively). In all, the enriched Levenshtein-distance similarity matrices covering the sequence, expression and functional miRNA similarities have effectively enhanced the performance for causal miRNA-disease association prediction.

Although LE-MDCAP has an acceptable performance in prioritizing causal miRNA-disease associations from a large number of general miRNA-disease associations, it still has clear limitations. First, a realistic limitation is that the disease prediction space is limited to diseases included in the causal miRNA-disease association dataset, resulting in prediction models that do not apply to new diseases without any known causal associations with miRNAs. The prediction performance of LF-MDCAP would improve with the amount of disease causal miRNA annotation data increasing in future work. Second, the AUROC of MDCAP for causal versus non-disease prediction is 0.928 and 0.925 in 10-fold cross-validation and independent testing, respectively, outperforming LE-MDCAP. To elevate the prediction accuracy of our model, we tried to combine the miRNA target information data, but this only resulted in a marginal performance improvement (Supplementary Materials Figure S1). In the future, the better construction of the disease semantic similarity matrix may further improve the performance. Third, designing score functions for causal miRNA-disease associations by accumulating works from the literature may also help to extract additional features for the causal miRNA-disease association prediction models in the future.

## 4. Materials and Methods

### 4.1. Human Causal miRNA-Disease Associations

The human causal miRNA-disease associations dataset was downloaded directly from HMDD v3.2 (http://www.cuilab.cn/hmdd/, accessed on 18 May 2021) [11]. To compare our method with Gao’s method [13], we use the same datasets as they did that contain 4228 experimentally verified causal associations between 535 miRNAs and 302 diseases. We constructed a *nm* × *nd* adjacency matrix, *MD*, to better represent the causal miRNA-disease associations, where nm and nd denote the number of miRNAs and diseases, respectively. Specifically, the element *MD*(*i*, *j*) is 1 if miRNA *m*(*i*) is confirmed to be causally associated with disease *d*(*j*); otherwise, it is 0.

### 4.2. MiRNAs Similarities

To more fully characterize the similarity of miRNAs, we introduced the Levenshtein-distance algorithm to measure the feature similarity between any two miRNAs. The Levenshtein distance, also known as the edit distance between strings, is defined as the minimum number of operations required to make two inputs equal. Thus, we obtain the following Equation (1):

(1) $$0\leq LD\prime \left(m_{1},m_{2}\right)\leq len\left(m_{1}\right)+len\left(m_{2}\right)$$

where *LD′* (*m*_1_, *m*_2_) represents the minimum editing cost of converting the miRNA *m*_1_ feature string to another miRNA *m*_2_ feature string, and *len* represents the length of miRNA feature string.

Therefore, the functional similarity of two miRNAs as *MS* (*m*_1_, *m*_2_) can be calculated as follows Equation (2):

(2) $$MS\left(m_{1},m_{2}\right)=1-\frac{LD\prime \left(m_{1},m_{2}\right)}{len\left(m_{1}\right)+len\left(m_{2}\right)}$$

Because only unilateral editing distance was considered here, the calculated *MS* (*m*_1_, *m*_2_) scores should range from 0.5 to 1. A larger score indicates that the two miRNA feature strings are more similar and therefore more likely to perform similar functions.

Instead of being simply an approach for measuring sequence similarity between miRNAs, Levenshtein distance was employed to established an enriched set of miRNA similarity matrixed covering the similarity in seed sequences, mature miRNA sequences, hairpin precursor sequences, expression levels and target pathways between miRNAs. First, miRNAs follow the base-pairing principle when binding to their target genes, and, more importantly, sequence features could be applied to all miRNAs without the bias reported in the literature. Therefore, we here used the sequence information of miRNAs as the primary proxy to probe their functions. The sequence data of miRNAs from the miRbase (http://www.mirbase.org/, version 22, accessed on 30 September 2021) [36] were collected, and the Levenshtein distance was utilized to measure the similarity of pre-miRNA sequences, mature miRNA sequences and seed sequences between any two miRNAs. Accordingly, three functional similarity matrices, namely *MS_SP_*, *MS_SM_* and *MS_SS_*, were obtained. The sequence-information-based miRNA similarity matrix, *MS_S_*, was obtained based on the weighted sum of the above three matrixes, where the weights of all scores sum to 1 and were optimized in steps of 0.05. We introduced the *MS_S_* obtained by combining different weights into the algorithm separately and finally selected the combined weight (*MS_S_ =* 0.05*MS_SP_ +* 0.05*MS_SM_ +* 0.9*MS_SS_*) due to its better AUROC value when the algorithm distinguished causal and non-causal miRNA-disease associations (Supplementary Materials Table S1). Second, as a typical class of non-coding RNAs, the expression profiles of miRNAs are often cell-type-specific, and the function of a miRNA is heavily dependent on what cell it is expressed by. For this reason, we obtained miRNAs expression data from Lorenzi’s study [37] for 137 cell types, followed by calculating miRNA expression similarity by Levenshtein distance to determine the functional similarity matrix, *MS_E_*—more specifically, by classifying the expression level of a miRNA in each cell type as A, B, C and D, according to the quantile allocation of its expression level across all miRNAs. We have also tried other configurations but find such a quantile allocation performed slightly better than others (Supplementary Materials Figure S3); the expression data for each miRNA can be depicted as an expression string of length 137 that were further used for the Levenshtein distance algorithm. Third, a straightforward description of functional similarities between miRNAs is to measure how their targeted biological processes and signaling pathways overlap. Accordingly, we downloaded the *p*-value data for miRNA pathway enrichment analysis results from the miRPathDB (https://mpd.bioinf.uni-sb.de/, version 2.0, accessed on 17 October 2021) [38] database and screened for pathways with at least three miRNAs with *p*-value < 0.05. We graded the *p*-values of the 1409 retained pathways in four levels: specifically, A represented *p*-values less than 0.05 and greater than 0.01, B represented *p*-values less than 0.01 and greater than 0.0001, C represented *p*-values less than 0.0001 and N indicated non-significant *p*-values greater than 0.05. Therefore, each miRNA is assigned a 1409-dimononal pathway string vector for subsequent Levenshtein-distance calculations, resulting in the functional similarity matrix, *MS_P_*, based on the miRNA target pathways.

In addition, according to previous studies [39,40], we also constructed the Gaussian interaction profile kernel similarity matrix, *GM*, for miRNAs as the baseline method for miRNA similarity. Together, we finally calculated four miRNA similarity matrices, i.e., sequence-based, *MS_S_*; expression-based, *MS_E_*; pathway-based, *MS_P_*; and the previous Gaussian interaction profile kernel similarity matrix, *GM*.

### 4.3. Disease Semantic Similarity

The widely applied Wang’s disease semantic similarity [41] was introduced, which is based on sematic topology relations between diseases as recorded in the Medical Subject Headings (MeSH) database (https://www.nlm.nih.gov/, accessed on 19 July 2020). In the MeSH system, the topology of disease can be described as a directed acyclic graph (DAG), i.e., *DAG_D_ =* (*D*, *T_D_*, *E_D_)*, where *T_D_* denotes the node set that includes the disease, *D,* and its ancestor diseases; and *E_D_* denotes the edge set of all relationships of *DAG_D_*. The contribution of disease, *d*, to the semantic value of disease, *D*, can be defined by the following Equation (3):

(3) {DD(D)=1 DD(d)=max{Δ∗DD(d′)∣d′∈ childen of d} if d≠D

In the above equation, Δ is the semantic contribution factor, which is usually set to 0.5 [42]. The semantic value *DC*(*D*) is given by integrating all contributions of the ancestral disease and the disease, *D*, Equation (4):

(4) $$DC\left(D\right)=\sum_{d\in T\left(D\right)}D_{D}\left(d\right)$$

Therefore, the semantic similarity of diseases *D_i_* and *D_j_* is calculated as follows Equation (5):

(5) $$DSS\left(D_{i},D_{j}\right)=\frac{\sum_{d\in T\left(D_{i}\right)\cap T\left(D_{j}\right)}\left(D_{D_{i}}\left(d\right)+D_{D_{j}}\left(d\right)\right)}{DC\left(D_{i}\right)+DC\left(D_{j}\right)}$$

It is obvious that diseases sharing most of the DAGs are more likely to have higher semantic similarity.

### 4.4. Matrix Decomposition

From the above, we obtained the causal miRNA-disease association matrix, *MD*; the disease semantic similarity matrix, *DS*; and four miRNA similarity matrices, namely *MS_S_*, *MS_E_*, *MS_P_* and *GM*. Next, we utilized the matrix decomposition algorithm proposed by Che et al. [20] to predict the causal association scores of miRNAs with diseases, respectively.

First, the initial projection vector of each miRNA and disease is given in a fixed *k* dimensional space, and their inner product is used to represent the causal association between them, as Equation (6):

(6) $$MD\prime =M^{T}D$$

where *M* and *D* are *k × m* and *k × d* matrices, respectively; *m* is the number of miRNAs; and *D* is the number of diseases. Thus, the causal miRNA-disease association problem can be thought of as minimizing the distance between *MD’* matrix and *MD* matrix of known causality by solving for the appropriate *M* and *D*. The objective function can be expressed as follows Equation (7):

(7) $$\min\sum_{MD_{i,j}=1}{\left(MD_{i,j}^{\prime }-MD_{i,j}\right)}^{2}$$

On the other hand, the *M* and *D* should also fit the known miRNA similarity matrices and disease semantic similarity matrix in the model, so another part of the objective function is as follows Equation (8):

(8) $$\min\lambda_{1}{\left\|MM^{T}-MS\right\|}_{F}^{2}+\lambda_{2}{\left\|DD^{T}-DS\right\|}_{F}^{2}$$

These two parts of objective function can be optimized together by using the iterative least square approach, which was specified in Che et al.’s original article [20].

### 4.5. Integrated Prediction Score of LE-MDCAP

The inner product of the calculated *M* and *D* yields a prediction association score matrix, *MD′= M ^T^D*. The four miRNA similarity matrices, namely *MS_S_*, *MS_E_*, *MS_P_* and *GM*, correspond to the predicted score matrices, namely *MD′_S_*, *MD′_E_*, *MD′_P_* and *MD′_G_*, respectively. The composite prediction score matrix, *MD′*, is obtained based on the weighted sum of the above four prediction scores, where the weights of all scores sum to 1 and have been optimized in steps of 0.05. Finally, we determine the integrated prediction score matrix, *MD′*, as *MD′* = 0.35*MD′_S_ +* 0.4 *MD′_E_ +* 0.15*MD′_P_ +* 0.1*MD′_G_* (Supplementary Materials Table S2).

### 4.6. Model Evaluation and Server Construction

To evaluate the prediction accuracy of LF-MDCAP, we also performed an independent test and 10-fold cross-validation. In terms of distinguishing causal from non-causal miRNA-disease associations, our model was compared with the MDCAP predictor. The prediction of LE-MDCAP was available as an online web server that was constructed with the HTML + PHP + Apache framework.

## Figures and Tables

**Table 1:** Top 15 miRNAs predicted by LF-MDCAP to be causally associated with diabetic retinopathy.

Rank	miRNA	Disease	Score	PMID
1	has-mir-21	Diabetic retinopathy	0.1040	32106367
2	has-mir-34a	Diabetic retinopathy	0.0650	33064974
3	has-mir-221	Diabetic retinopathy	0.0562	32648125
4	has-mir-126	Diabetic retinopathy	0.0560	31608649
5	has-mir-106b	Diabetic retinopathy	0.0502	NA
6	hsa-mir-155	Diabetic retinopathy	0.0453	NA
7	hsa-mir-503	Diabetic retinopathy	0.0446	NA
8	hsa-mir-125b	Diabetic retinopathy	0.0431	30988072
9	hsa-mir-590	Diabetic retinopathy	0.0428	31618425
10	hsa-mir-223	Diabetic retinopathy	0.0413	31415795
11	hsa-mir-330	Diabetic retinopathy	0.0406	NA
12	hsa-mir-145	Diabetic retinopathy	0.0406	NA
13	hsa-mir-222	Diabetic retinopathy	0.0405	NA
14	hsa-mir-16	Diabetic retinopathy	0.0396	NA
15	hsa-mir-18a	Diabetic retinopathy	0.0393	32210827

**Table 2:** Top 15 diseases predicted by LF-MDCAP to be causally associated with has-mir-361.

Rank	Disease	miRNA	Score	PMID
1	Breast Neoplasms	hsa-mir-361	0.157	32092817
2	Prostatic Neoplasms	hsa-mir-361	0.149	31957820
3	Stomach Neoplasms	hsa-mir-361	0.141	31850945
4	Carcinoma, Non-Small-Cell Lung	hsa-mir-361	0.128	32197208
5	Glioblastoma	hsa-mir-361	0.112	32770454
6	Osteosarcoma	hsa-mir-361	0.106	34716310
7	Lung Neoplasms	hsa-mir-361	0.089	NA
8	Uterine Cervical Neoplasms	hsa-mir-361	0.088	33063235
9	Urinary Bladder Neoplasms	hsa-mir-361	0.078	NA
10	Carcinoma, Renal Cell	hsa-mir-361	0.075	34516333
11	Melanoma	hsa-mir-361	0.074	NA
12	Ovarian Neoplasms	hsa-mir-361	0.069	33500694
13	Inflammation	hsa-mir-361	0.067	NA
14	Atherosclerosis	hsa-mir-361	0.067	NA
15	Endometrial Neoplasms	hsa-mir-361	0.058	31287002

## Data Availability

LE-MDCAP was available at http://www.rnanut.net/LEMDCAP/. The dataset and prediction results are also available at https://github.com/bioinfohy/LE-MDCAP/.

## Supplementary Materials

All data are available online at https://www.mdpi.com/article/10.3390/ijms222413607/s1.

## References

[1] Wang H., Wang H., Duan X., Liu C., Li Z. (2017). Digital quantitative analysis of microRNA in single cell based on ligation-depended polymerase colony (Polony). Biosens. Bioelectron..

[2] Ambros V. (2004). The functions of animal microRNAs. Nature.

[3] Esteller M. (2011). Non-coding RNAs in human disease. Nat. Rev. Genet..

[4] Gebert L.F.R., MacRae I.J. (2019). Regulation of microRNA function in animals. Nat. Rev. Mol. Cell Biol..

[5] Meola N., Gennarino V., Banfi S. (2009). microRNAs and genetic diseases. Pathogenetics.

[6] Yang B., Lin H., Xiao J., Lu Y., Luo X., Li B., Zhang Y., Xu C., Bai Y., Wang H. (2007). The muscle-specific microRNA miR-1 regulates cardiac arrhythmogenic potential by targeting GJA1 and KCNJ2. Nat. Med..

[7] Zhao Y., Ransom J.F., Li A., Vedantham V., von Drehle M., Muth A.N., Tsuchihashi T., McManus M.T., Schwartz R.J., Srivastava D. (2007). Dysregulation of Cardiogenesis, Cardiac Conduction, and Cell Cycle in Mice Lacking miRNA-1-2. Cell.

[8] Png K.J., Yoshida M., Zhang X.H.F., Shu W., Lee H., Rimner A., Chan T.A., Comen E., Andrade V.P., Kim S.W. (2011). MicroRNA-335 inhibits tumor reinitiation and is silenced through genetic and epigenetic mechanisms in human breast cancer. Genes Dev..

[9] Valastyan S., Reinhardt F., Benaich N., Calogrias D., Szász A.M., Wang Z.C., Brock J.E., Richardson A.L., Weinberg R.A. (2009). A Pleiotropically Acting MicroRNA, miR-31, Inhibits Breast Cancer Metastasis. Cell.

[10] Williams A.H., Valdez G., Moresi V., Qi X., McAnally J., Elliott J.L., Bassel-Duby R., Sanes J.R., Olson E.N. (2009). MicroRNA-206 delays ALS progression and promotes regeneration of neuromuscular synapses in mice. Science.

[11] Huang Z., Shi J., Gao Y., Cui C., Zhang S., Li J., Zhou Y., Cui Q. (2019). HMDD v3.0: A database for experimentally supported human microRNA-disease associations. Nucleic Acids Res..

[12] Gao Y., Jia K., Shi J., Zhou Y., Cui Q. (2019). A Computational Model to Predict the Causal miRNAs for Diseases. Front. Genet..

[13] Huang Z., Liu L., Gao Y., Shi J., Cui Q., Li J., Zhou Y. (2019). Benchmark of computational methods for predicting microRNA-disease associations. Genome Biol..

[14] Chen X., Xie D., Zhao Q., You Z.H. (2019). MicroRNAs and complex diseases: From experimental results to computational models. Brief. Bioinform..

[15] Wang L., You Z.H., Chen X., Li Y.M., Dong Y.N., Li L.P., Zheng K. (2019). LMTRDA: Using logistic model tree to predict MiRNA-disease associations by fusing multisource information of sequences and similarities. PLoS Comput. Biol..

[16] Yu S.P., Liang C., Xiao Q., Li G.H., Ding P.J., Luo J.W. (2019). MCLPMDA: A novel method for miRNA-disease association prediction based on matrix completion and label propagation. J. Cell. Mol. Med..

[17] Li G., Luo J., Xiao Q., Liang C., Ding P. (2018). Predicting microRNA-disease associations using label propagation based on linear neighborhood similarity. J. Biomed. Inform..

[18] Qu Y., Zhang H., Liang C., Ding P., Luo J. (2018). SNMDA: A novel method for predicting microRNA-disease associations based on sparse neighbourhood. J. Cell. Mol. Med..

[19] Chen X., Huang L., Xie D., Zhao Q. (2018). EGBMMDA: Extreme gradient boosting machine for MiRNA-disease association prediction. Cell Death Dis..

[20] Che K., Guo M., Wang C., Liu X., Chen X. (2019). Predicting MiRNA-disease association by latent feature extraction with positive samples. Genes.

[21] Cheung N., Mitchell P., Wong T.Y. (2010). Diabetic retinopathy. Lancet.

[22] Dong Y., Wan G., Peng G., Yan P., Qian C., Li F. (2020). Long non-coding RNA XIST regulates hyperglycemia-associated apoptosis and migration in human retinal pigment epithelial cells. Biomed. Pharmacother..

[23] Ji Q., Han J., Wang L., Liu J., Dong Y., Zhu K., Shi L. (2020). MicroRNA-34a promotes apoptosis of retinal vascular endothelial cells by targeting SIRT1 in rats with diabetic retinopathy. Cell Cycle.

[24] Wang C., Lin Y., Fu Y., Zhang D., Xin Y. (2020). MiR-221-3p regulates the microvascular dysfunction in diabetic retinopathy by targeting TIMP3. Pflug. Arch. Eur. J. Physiol..

[25] Chen X., Yu X., Li X., Li L., Li F., Guo T., Guan C., Miao L., Cao G. (2020). MiR-126 targets IL-17A to enhance proliferation and inhibit apoptosis in high-glucose-induced human retinal endothelial cells. Biochem. Cell Biol..

[26] Xu D., Dong P., Xiong Y., Yue J., Ihira K., Konno Y., Kobayashi N., Todo Y., Watari H. (2019). MicroRNA-361: A multifaceted player regulating tumor aggressiveness and tumor microenvironment formation. Cancers.

[27] Wang K., Liu C.Y., Zhang X.J., Feng C., Zhou L.Y., Zhao Y., Li P.F. (2015). MiR-361-regulated prohibitin inhibits mitochondrial fission and apoptosis and protects heart from ischemia injury. Cell Death Differ..

[28] Hua B., Li Y., Yang X., Niu X., Zhao Y., Zhu X. (2020). MicroRNA-361-3p promotes human breast cancer cell viability by inhibiting the E2F1/P73 signalling pathway. Biomed. Pharmacother..

[29] Qian B., Zhang D., Tao R., Yu G., Jia B., Ye K., Ma L., Wan S., Wu W. (2020). MiR-361-5p exerts tumor-suppressing functions in gastric carcinoma by targeting syndecan-binding protein. Anticancer Drugs.

[30] Li H.Y., Jiang F.Q., Chu L., Wei X. (2020). Long non-coding RNA BLACAT1 inhibits prostate cancer cell proliferation through sponging miR-361. Eur. Rev. Med. Pharmacol. Sci..

[31] Qi G., Li L. (2020). Long non-coding RNA PVT1 contributes to cell growth and metastasis in non-small-cell lung cancer by regulating miR-361-3p/SOX9 axis and activating Wnt/β-catenin signaling pathway. Biomed. Pharmacother..

[32] Zhou C., Jiang X., Liang A., Zhu R., Yang Y., Zhong L., Wan D. (2020). COX10-AS1 Facilitates Cell Proliferation and Inhibits Cell Apoptosis in Glioblastoma Cells at Post-Transcription Level. Neurochem. Res..

[33] Zhang H.D., Jiang L.H., Sun D.W., Hou J.C., Ji Z.L. (2018). CircRNA: A novel type of biomarker for cancer. Breast Cancer.

[34] Kai K., Dittmar R.L., Sen S. (2018). Secretory microRNAs as biomarkers of cancer. Semin. Cell Dev. Biol..

[35] Wiedrick J.T., Phillips J.I., Lusardi T.A., McFarland T.J., Lind B., Sandau U.S., Harrington C.A., Lapidus J.A., Galasko D.R., Quinn J.F. (2019). Validation of MicroRNA Biomarkers for Alzheimer’s Disease in Human Cerebrospinal Fluid. J. Alzheimer’s Dis..

[36] Kozomara A., Birgaoanu M., Griffiths-Jones S. (2019). MiRBase: From microRNA sequences to function. Nucleic Acids Res..

[37] Lorenzi L., Chiu H.S., Avila Cobos F., Gross S., Volders P.J., Cannoodt R., Nuytens J., Vanderheyden K., Anckaert J., Lefever S. (2021). The RNA Atlas expands the catalog of human non-coding RNAs. Nat. Biotechnol..

[38] Kehl T., Kern F., Backes C., Fehlmann T., Stöckel D., Meese E., Lenhof H.P., Keller A. (2020). MiRPathDB 2.0: A novel release of the miRNA Pathway Dictionary Database. Nucleic Acids Res..

[39] Van Laarhoven T., Nabuurs S.B., Marchiori E. (2011). Gaussian interaction profile kernels for predicting drug-target interaction. Bioinformatics.

[40] Lu M., Zhang Q., Deng M., Miao J., Guo Y., Gao W., Cui Q. (2008). An analysis of human microRNA and disease associations. PLoS ONE.

[41] Wang D., Wang J., Lu M., Song F., Cui Q. (2010). Inferring the human microRNA functional similarity and functional network based on microRNA-associated diseases. Bioinformatics.

[42] Sun D., Li A., Feng H., Wang M. (2016). NTSMDA: Prediction of miRNA-disease associations by integrating network topological similarity. Mol. Biosyst..

