# Supplementary material for: LE-MDCAP: A Computational Model to Prioritize Causal miRNA-Disease Associations
Source: Int J Mol Sci. 2021 Dec 19;22(24):13607. doi: 10.3390/ijms222413607 (PMC8706837; doi:10.3390/ijms222413607)
Supplement: Supplementary file 1 [file ijms-22-13607-s001.zip › ijms-1487402-supplementary.pdf]

Supplementary Materials

LE-MDCAP: A computational model to prioritize causal miRNA-disease associations

Supplementary Table

Supplementary Table S1. Performance of the algorithm under different weight integration conditions of MS_{SP}, MS_{SM}, and MS_{SS}

MS_{SP}	MS_{SM}	MS_{SS}	AUROC (causal vs. non-causal)	AUROC (causal vs. non-disease)
0.05	0.05	0.9	0.81727	0.90351
0.1	0.05	0.85	0.81718	0.90354
0.05	0.1	0.85	0.81714	0.90357
0.15	0.05	0.8	0.81711	0.90358
0.1	0.1	0.8	0.81707	0.90365
0.2	0.05	0.75	0.81704	0.90370
0.05	0.15	0.8	0.81702	0.90369
0.15	0.1	0.75	0.81699	0.90370
0.25	0.05	0.7	0.81697	0.90378
0.1	0.15	0.75	0.81694	0.90377

Supplementary Table S2. Performance of the algorithm under different weight integration conditions of MD'_S, MD'_E, MD'_P, and MD'_G.

MD'_S	MD'_E	MD'_P	MD'_G	AUROC (causal vs. non-disease)	AUROC (causal vs. non-causal)
0.35	0.4	0.15	0.1	0.9055169	0.8203798
0.35	0.35	0.2	0.1	0.9055168	0.820158
0.35	0.4	0.2	0.05	0.9055163	0.819574
0.3	0.4	0.2	0.1	0.9055139	0.820258
0.4	0.4	0.5	0.05	0.905512	0.819696
0.4	0.35	0.15	0.1	0.905512	0.820281
0.3	0.35	0.2	0.15	0.905512	0.820828
0.35	0.35	0.15	0.15	0.905505	0.82095
0.3	0.4	0.25	0.05	0.905503	0.819455
0.3	0.35	0.25	0.1	0.905501	0.820031

Supplementary Figures

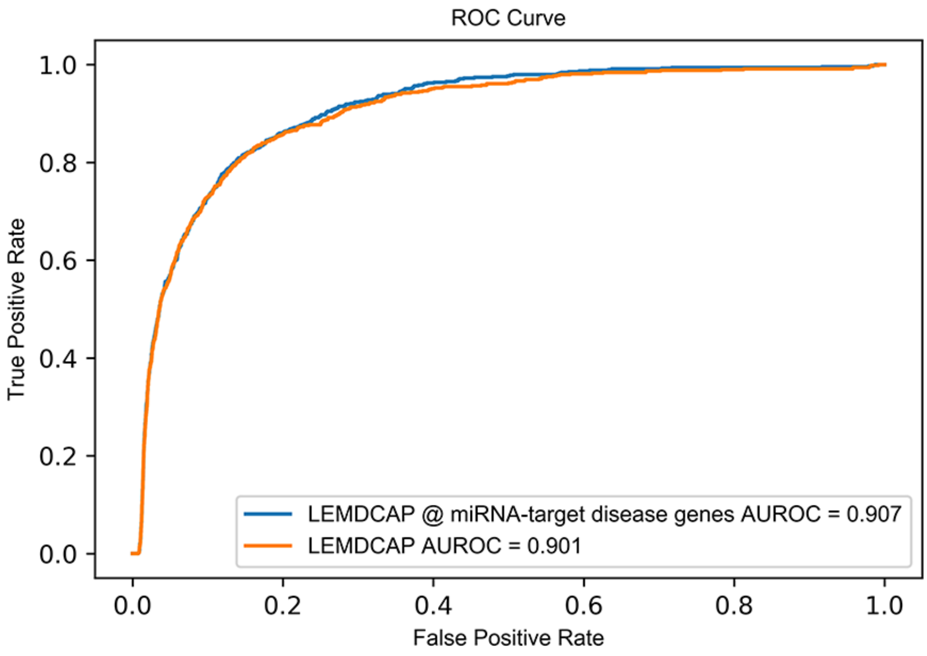

Supplementary Figure S1. Performance of LE-MDCAP after adding miRNA-target disease gene feature. ROC curve per-formed by modified LE-MDCAP in distinguishing causal miRNA-disease associations from the non-causal miRNA-disease associations.

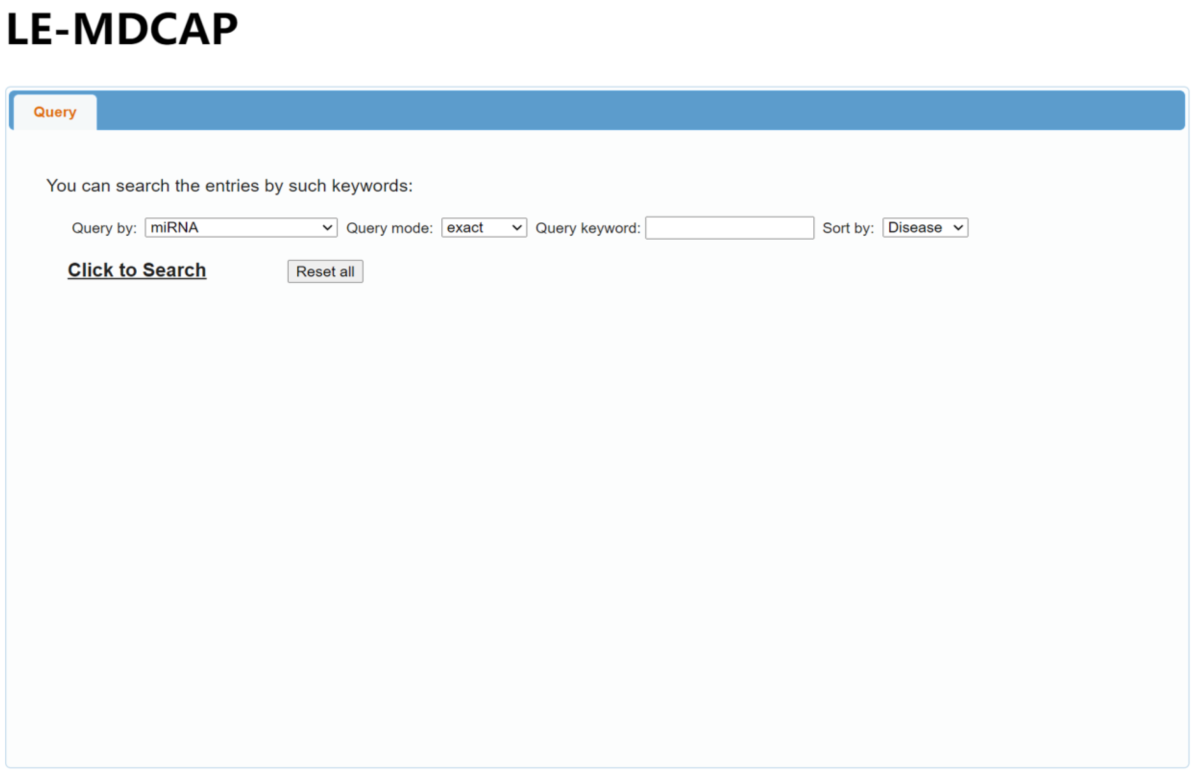

Supplementary Figure S2. The query interface of LE-MDCAP server.

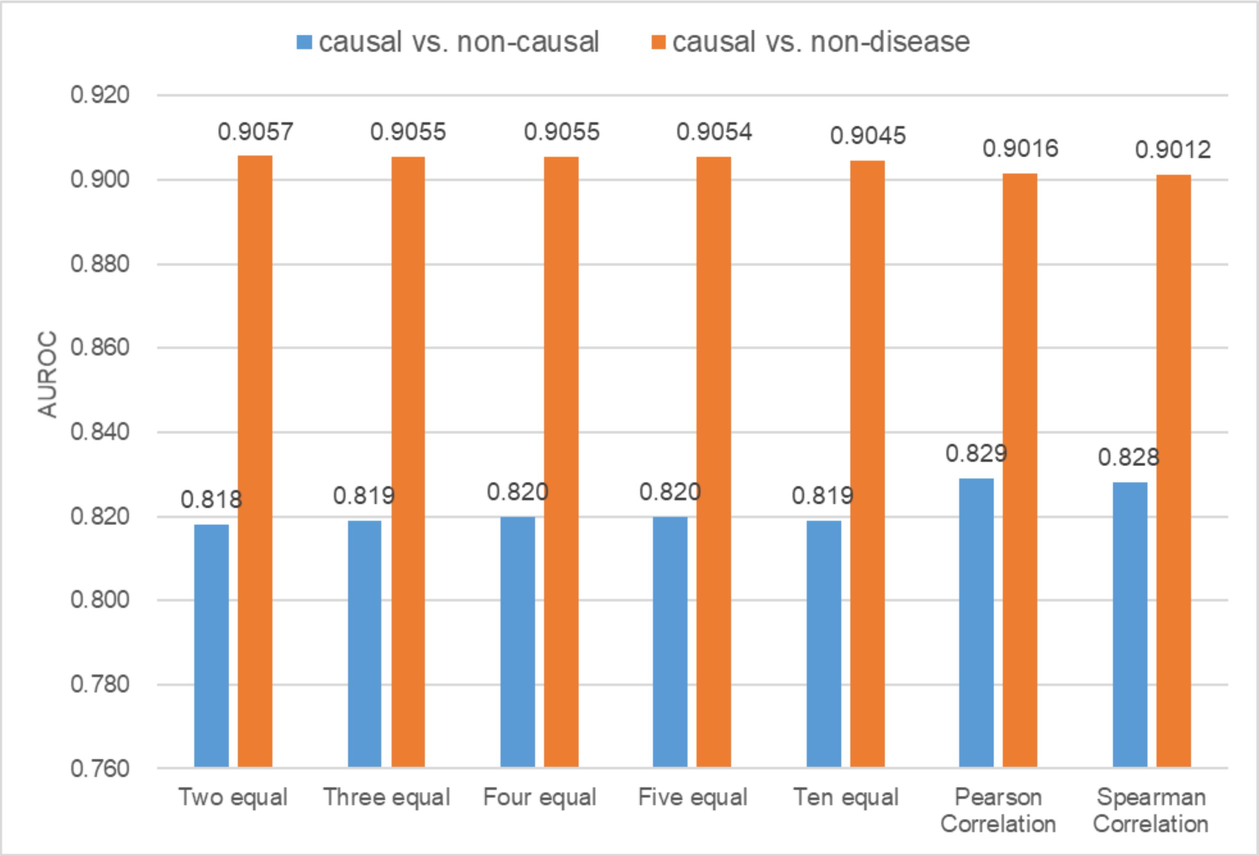

Supplementary Figure S3. Performance of LE-MDCAP under conditions of different classification of miRNA expression levels.
